# Quantification of gamma-secretase modulation differentiates inhibitor compound selectivity between two substrates Notch and amyloid precursor protein

**DOI:** 10.1186/1756-6606-1-15

**Published:** 2008-11-04

**Authors:** Ting Yang, Dilyara Arslanova, Yongli Gu, Corinne Augelli-Szafran, Weiming Xia

**Affiliations:** 1 Center for Neurologic Diseases, Department of Neurology, Brigham and Women's Hospital, Harvard Medical School, Harvard University, Boston, MA, USA

## Abstract

**Background:**

Deposition of amyloid-β protein (Aβ) is a major pathological hallmark of Alzheimer's disease (AD). Aβ is generated from γ-secretase cleavage of amyloid precursor protein (APP). In addition to APP, γ-secretase also cleaves other type I integral membrane proteins, including the Notch receptor, a key molecule involved in embryonic development.

**Results:**

To explore selective γ-secretase inhibitors, a combination of five methods was used to systematically determine these inhibitors' profiles on the γ-secretase cleavage of APP and Notch. When two potent γ-secretase inhibitors, compound E (cpd E) and DAPT, were used in a conventional *in vitro *γ-secretase activity assay, cpd E completely blocked Aβ generation from the cleavage of substrate APP C100, but only had a minor effect on Notch cleavage and NICD generation. Next, cpd E and DAPT were applied to HEK293 cells expressing a truncated Notch substrate NotchΔE. Both cpd E and DAPT were more potent in blocking Aβ generation than NICD generation. Third, a reporter construct was created that carried the NICD targeting promoter with three Su(H) binding sequences followed by the luciferase gene. We found that the inhibition of NICD generation by cpd E and DAPT was consistent with the reduced expression of luciferase gene driven by this Notch targeting promoter. Fourth, levels of "Notch-Aβ-like" (Nβ*) peptide derived from two previously reported chimeric APP with its transmembrane domain or the juxtamembrane portion replaced by the Notch sequence were quantified. Measurement of Nβ* peptides by ELISA confirmed that EC_50_'s of cpd E were much higher for Nβ* than Aβ. Finally, the expression levels of Notch target gene *her6 *in cpd E or DAPT-treated zebrafish were correlated with the degree of tail curvature due to defective somitogenesis, a well characterized Notch phenotype in zebrafish.

**Conclusion:**

Our ELISA-based quantification of Aβ and Nβ* in combination with the test in zebrafish provides a novel approach for efficient cell-based screening and *in vivo *validation of APP selective γ-secretase inhibitors.

## Background

Genetic and neuropathologic evidence suggests that Alzheimer's disease (AD) is caused partly by the overproduction and lack of clearance of the amyloid β peptide (Aβ) [1]. This Aβ peptide is generated by sequential cleavages of the amyloid precursor protein (APP) by β-secretase, which generates a 12 kDa C-terminal stub of APP (C99), and by γ-secretase to yield two major species of Aβ that end at residue 40 (Aβ_40_) or 42 (Aβ_42_) [2,3]. In addition to cleaving APP, γ-secretase also mediates the final proteolytic cleavage of the Notch receptor [4,5]. Notch signaling is critical to a wide variety of cell fate determinations during embryonic development as well as throughout adulthood. After ectodomain shedding, the remaining membrane-bound C-terminal stub is cleaved by γ-secretase to release the Notch-1-β peptide (Nβ, similar to amyloid β peptide from APP) and the Notch IntraCellular Domain (NICD). NICD is subsequently translocated to the nucleus where it regulates gene expression [5-7].

There are about 50 γ-secretase substrates in addition to APP and Notch that include DCC [8], ErbB-4 [9,10], E- and N-cadherin [11,12], CD44 [13,14], LRP [15], Nectin1α [16], Delta and Jagged [17], Glutamate Receptor Subunit 3 [18], APLP1 and APLP2 [19-21], p75 Neurotrophin Receptor [22], Syndecan3 [23], Colony Stimulating factor-1 [24] and Interleukin-1 Receptor II [25]. All of these substrates are type I membrane proteins and have diverse functions, including transcriptional regulation, cell-cell adhesion, regulation of ion conductance, and neurotrophin signaling. The cleavage of these proteins can be blocked by reported γ-secretase inhibitors and are fully dependent on each γ-secretase component [26].

γ-Secretase is composed of presenilin 1 (PS1), anterior pharynx defective-1 (Aph-1), presenilin enhancer-2 (Pen-2), and nicastrin (Nct). PS1 carries the catalytic site of γ-secretase, as we have demonstrated that a mutation of two critical aspartate (Asp) residues abrogates enzymatic activity [27]. Nicastrin is required for γ-secretase activity [28-35] and is an important component in the complex, possibly functioning as the receptor for different substrates [36]. Genetic screens further revealed the *aph-1 *gene and the *pen-2 *gene that encodes two essential components of the γ-secretase complex [37,30,38]; overexpression of all four components results in increased γ-secretase activity, both in mammalian cells [39-44] and in yeast [45].

Among all reported γ-secretase inhibitors, transition-state analogues prevent Aβ generation and bind directly to PS1 and PS2 [46,47]. Most reported γ-secretase inhibitors specifically block the cleavage at both sites in APP and Notch without differentiating between the two substrates. It has been reported that a subset of NSAIDS (nonsteroidal anti-inflammatory drugs) that include ibuprofen, indomethacin and sulindac sulphide, specifically block the cleavage of the γ-secretase substrates at the middle of transmembrane domain (TMD) without affecting the generation of the intracellular domains (ICDs) of several type I transmembrane proteins that include APP, ErbB-4, and Notch [48]. These NSAIDs directly modulate γ-secretase complex and become a part of a new class of γ-secretase modulators [49-54]. Another γ-secretase modulator is Gleevec that has been approved for the treatment of chronic myeloid leukemia and gastrointestinal stromal tumors. In addition to Gleevec binding to Abelson leukemia (Abl) tyrosine kinase, it also was shown to selectively inhibit APP cleavage and Aβ production without affecting Notch cleavage at the concentration of 10 μM [55].

Two potent γ-secretase inhibitors, DAPT and compound E (cpd E), show a range of IC_50 _values in blocking γ-secretase activity in both *in vitro *and cell-based assays. For cpd E, the IC_50 _for NICD and Aβ generation in cultured cells was found as low as 1.7 nM [56] and 0.3 nM [57], respectively. De novo Aβ and AICD generation *in vitro *was inhibited by DAPT with IC_50 _values ranging from 10–100 nM [58,59]. A direct comparison of NICD and AICD levels in an *in vitro *γ-secretase activity assay showed a partial inhibition of NICD generation by DAPT at 50 nM, and AICD at 100 nM [60]. Different assay systems were implemented in these various studies to measure the IC_50 _values of the γ-secretase inhibitors. Since there were a variety of assays used, it was difficult to compare the potency toward the cleavage of APP and Notch among different systems.

The current study combined five assay methods and systematically determined the pharmacological profile of cpd E and DAPT on γ-secretase cleavage of APP and Notch. This approach includes the measurements of the potency of γ-secretase inhibitors and their effect on the inhibition of the γ-secretase activity *in vitro*, NICD generation, NICD downstream transcription activation, cleavage of APP/Notch chimeric substrates, and Notch downstream target gene expression in zebrafish. Previous studies showed that treating zebrafish with DAPT at the late blastula stage caused defects in somitogenesis and neurogenesis [61]. Similarities have been observed between DAPT-treated embryos and previously reported zebrafish Notch pathway mutants like *bea, des, aei*, and *wit *[62,63]. The increased neurogenesis in DAPT-treated embryos can be reduced by microinjecting NICD mRNA. Interestingly, defective somitogenesis was not observed in zebrafish embryos that were treated with the Aβ-lowering JLK non-peptidic isocoumarin inhibitors [64]. In this study, the expression levels of Notch target gene *her 6 *were correlated to the phenotypes that were observed in the embryos treated with DAPT and cpd E. This provided an *in vivo *system to test the effect of γ-secretase inhibitors on Notch signaling in a whole vertebrate animal.

## Results

### Low concentration of compound E selectively blocks Aβ production with minimum effect on NICD generation *in vitro*

**Figure 1:**
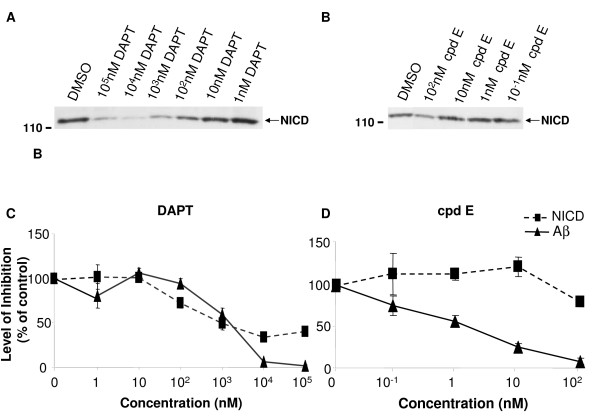
**Generation of NICD and Aβ from purified APP and Notch substrate C100 and N100 in an in vitro γ-secretase activity assay**. The *E. coli *generated APP- and Notch-based, 100-residue γ-secretase substrates C100-Flag and N100-Flag were mixed with the membrane vesicles solubilized in CHAPSO after DMSO or compounds were added. The mixture was incubated at 37°C for 4 hours. A. A dose-dependent inhibition of NICD generation by DAPT. Generation of NICD was detected by Western blot (WB) with antibody 1744 specifically recognizing N-terminus of NICD. B. Generation of NICD was inhibited in the presence of 100 nM of cpd E. C. Levels of NICD determined by WB were quantified by densitometry (dotted line). Levels of Aβ generated from the γ-secretase cleavage of C100 in the presence of DAPT were determined by ELISA (solid line). Comparison of NICD and Aβ generation in the presence of DAPT suggests that high concentrations of DAPT were more potent in blocking Aβ than NICD generation. D. NICD (dotted line) and Aβ (solid line) generation in the presence of cpd E were compared. Cpd E inhibited Aβ generation with an IC_50 _of 1 nM and is more potent in inhibiting Aβ than NICD.

To characterize the direct effect of two γ-secretase inhibitors cpd E and DAPT on APP/Notch cleavage, a conventional *in vitro *γ-secretase assay to quantify their inhibitory potency was used [58,65,66]. The incubation of γ-secretase complex with purified substrates at 37°C for 4 hr was followed by Western Blot (WB) to determine the quantity of newly generated NICD. A newly generated band that corresponds to the predicted size of the NICD-Flag was detected (Fig. 1A and 1B). A clear reduction of NICD generation in samples containing DAPT (Fig. 1A) or cpd E (Fig. 1B) was found, and the reduction was dose dependent.

The same preparation of γ-secretase complex was mixed with C100Flag followed by ELISA to quantify the levels of newly generated Aβ. As expected, both DAPT (Fig. 1C) and cpd E (Fig. 1D) blocked γ-secretase cleavage of APP C100Flag and caused a dose-dependent reduction of Aβ production.

Close comparison of the inhibition profile of cpd E and DAPT on Aβ and NICD generation revealed a divergence in their potencies. Low concentrations of DAPT did not show much difference in inhibiting NICD and Aβ generation, but 10 and 100 μM of DAPT blocked ~60% of NICD generation compared to a complete depletion of Aβ production (Fig. 1C). While 100 nM of cpd E could almost deplete any Aβ generation from substrate APP C100, its effect on NICD was much less obvious (Fig. 1D). There was only minor reduction of NICD levels compared to DMSO controls. This led to the speculation that certain γ-secretase inhibitors may specifically inhibit APP at a particular range of doses that have minimum effect on Notch signaling.

### Compound E and DAPT differentially inhibit Aβ and NICD generation in cultured cells

**Figure 2:**
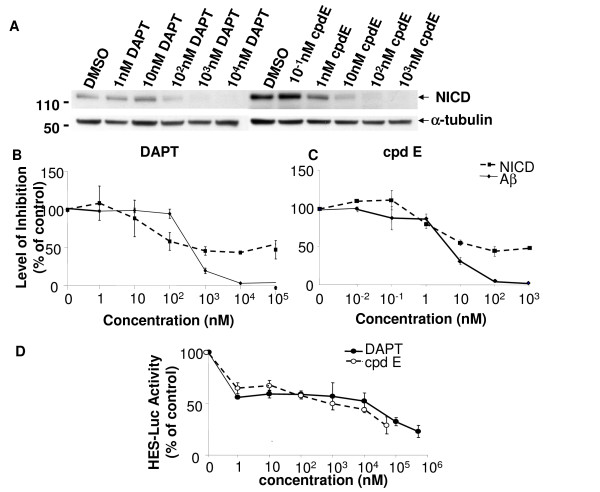
**Generation of NICD and Aβ from NotchΔE and APP expressing cells**. A. Twenty four hrs after the construct carrying NotchΔE was transfected into APP expressing HEK293 cells, cells were treated with DAPT or cpd E for 8 hr and lysed for WB with antibody 1744 to specifically detect the N-terminus of NICD. Bottom panel, the antibody against α-tubulin was applied to normalize the amounts of lysates used for WB. B. Levels of NICD determined by WB were quantified by densitometry (dotted line). Levels of Aβ generated from the γ-secretase cleavage of APP in the presence of DAPT were determined by ELISA. Comparison of NICD and Aβ generation in the presence of DAPT suggests that high concentrations of DAPT had a greater inhibition of Aβ than NICD. C. NICD (dotted line) and Aβ (solid line) production from cpd E-treated cells were compared. Cpd E inhibited Aβ generation with an IC_50 _of ~8 nM, and it shows a greater inhibition of Aβ than NICD. D. A luciferase reporter construct driven by HES1 promoter was transfected into HEK293 cells followed by treatment with cpd E or DAPT. Both γ-secretase inhibitors blocked transcriptional activation of NICD dependent luciferase activity.

Since many compounds could behave differently *in vitro *versus in culture cells, cpd E and DAPT were tested in cultured cells (Fig. 2). HEK293 cells stably expressing Swedish mutant APP were transiently transfected with NotchΔE, a truncated Notch construct that is readily cleaved by the γ-secretase to generate NICD for downstream signaling transduction [67]. NotchΔE expressing cells were treated with increasing concentrations of DAPT or cpd E. Cell lysates were subjected to WB for measuring the generation of NICD (Fig. 2A), and conditioned media were collected for Aβ measurement by ELISA (Fig. 2B,C).

Semi-quantification of NICD levels was detected by WB, and the inhibition profile of DAPT (Fig. 2B) and cpd E (Fig. 2C) were compared on NICD and Aβ generation in cultured cells. It was found that high doses of DAPT and cpd E could not completely eliminate NICD generation in cultured cells. This was in contrast to Aβ levels that were efficiently reduced to almost undetectable levels. Since Notch signaling and levels of NICD can be examined by quantifying the expression of the Notch target gene, a Hes-1 reporter construct (Hes-Luc) was generated by insertion of three Su(H) binding sequences in the pGL3-pro luciferase reporter vector. Hes-Luc and NotchΔE were transiently transfected into HEK293 cells, and transfected cells were treated with different concentrations of cpd E or DAPT. Consistent with the levels of NICD that was freshly generated in cultured cells, luciferase activities were inhibited by relatively high doses of cpd E and DAPT. At the concentrations of cpd E and DAPT that completely blocked Aβ generation (Fig. 2B and 2C), about 50% luciferase activities remained, i.e., inhibition of NICD generation was less efficient compared to Aβ blockage (Fig. 2D).

### A chimeric APP-Notch ELISA differentiates cpd E in inhibiting APP versus chimeric APP-Notch

Two cDNA constructs expressing chimeric APP and Notch were previously reported to generate chimeric "Notch-Aβ-like" (Nβ*) peptide [68]. One construct has its transmembrane domain (TMD) replaced by the Notch TMD (APP-m-NOTCH) and the other with the juxtamembrane portion of the APP ectodomain (the α-secretase cleavage region) replaced by the corresponding sequence in Notch (APP-α-NOTCH) [68]. Taking advantage of different combinations of ELISA antibodies (see Methods), effects of cpd E on the generation of Aβ and Nβ* peptides from these chimeric APP-Notch substrates were quantified by ELISA.

**Figure 3:**
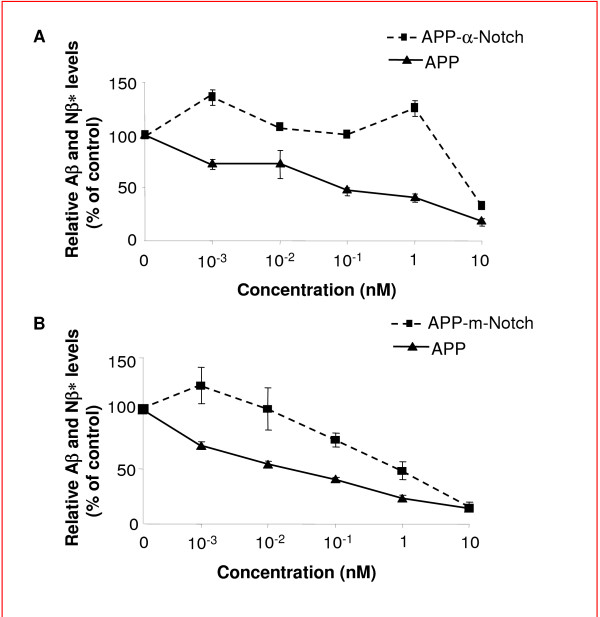
Generation of Aβ and Nβ* from chimeric APP-Notch expressing cells. A. The juxtamembrane portion of the APP ectodomain (the α-secretase cleavage region) was replaced by the corresponding sequence in Notch (α-NOTCH). Levels of Nβ* in the media from cells expressing APP-α-Notch (dotted line) and levels of Aβ from APP expressing cells (solid line) were determined by ELISA. B. A chimeric cDNA constructs express APP with its transmembrane domain (TMD) replaced by the Notch TMD (APP-m-NOTCH). Levels of Aβ (solid line) and Nβ* (dotted line) were determined by ELISA.

Individual construct APP, APP-α-Notch or APP-m-Notch was transiently transfected into HEK293 cells. These chimeric protein expressing cells were treated with cpd E, and the levels of Aβ and Nβ* were measured by ELISA. Again, it was found that the effective concentration (EC) for inhibiting 50% of Aβ production by cpd E was less than 0.1 nM, but the EC_50 _for Nβ* from α-Notch was at ~8 nM (Fig. 3A). Similar results were obtained when m-Notch was expressed in HEK293 cells. At least two magnitude of difference was observed, with EC_50 _for cpd E was ~0.03 nM for APP, compared to EC_50 _for Nβ* at ~1 nM (Fig. 3B).

### Defective zebrafish phenotypes illustrated inhibition of Notch signaling

Measurements of *in vitro *γ-secretase activity and cell-based Aβ/NICD generation have shown different inhibition potencies. To examine the inhibitory effect *in vivo*, zebrafish embryos were treated with DAPT or cpd E. Because different γ-secretase inhibitors may impact various metabolic pathways in zebrafish embryos, especially during development, the phenotypes of zebrafish embryos treated with high concentrations of DAPT and cpd E were compared. The major phenotype we examined was curved tail caused by defective somitogenesis.

**Figure 4:**
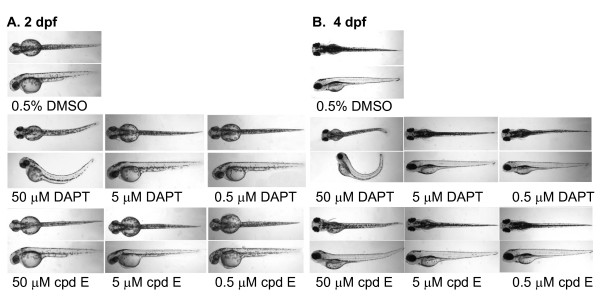
**Treatment of zebrafish embryos with DAPT causes curved tails**. A. A stock of DAPT or cpd E in DMSO was diluted in embryo medium, and increasing concentrations of DAPT or cpd E were applied to de-chorionated zebrafish embryos incubated at 28°C from 24 hpf to 48 hpf. Control embryos were mock-treated with embryo medium containing the same concentration of DMSO. Treatment of zebrafish embryos with 50 μM DAPT caused curved trunk and tails. B. DAPT- or cpd E-treated embryos were kept until 4 dpf, and images were acquired at 40 × magnification.

Morphological alteration in DAPT- or cpd E-treated embryos was compared and correlated to the somitogenesis associated with the inhibition of Notch signaling. The treated embryos were examined using a stereomicroscope and it was found that embryos treated with 50 μM DAPT had a much shorter and curved tail, compared to control DMSO-treated embryos (Fig. 4A). The curvature was obvious when a lateral view of zebrafish was obtained. Cpd E, on the other hand, did not show any curvature when treated at 50 μM. Because the EC_50 _values for DAPT and cpd E to reduce NICD generation in cultured cells were ~1000 nM and 10 nM, respectively (Fig. 2B and 2C), 50 μM of DAPT and cpd E were chosen as the highest concentrations for the treatment.

When embryos were kept for four days, embryos treated with 50 μM DAPT continued to show the curvature of the tails (Fig. 4B). DMSO-treated embryos exhibited normal morphology with straight trunk and tail. Cpd E had a minor effect on embryo morphology, and the embryos maintained straight trunk and tails (Fig. 4B).

### Expression of Notch target gene her6 correlates with the phenotypes of zebrafish treated with γ-secretase inhibitors

**Figure 5:**
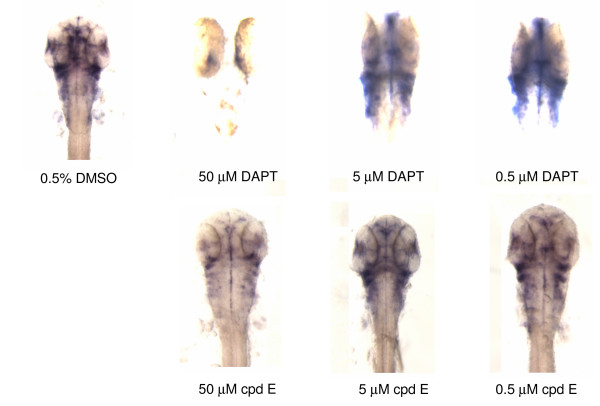
**Expression levels of Notch target gene *her6 *are consistent with the curvature phenotype**. Increasing concentrations of DAPT or cpd E were applied to de-chorionated zebrafish embryos from 24 hpf until 48 hpf, and *in situ *hybridization of compound-treated embryos was carried out at 48 hpf using the *her6 *probe. At least 10 to 20 embryos were examined for each experiment. Images were taken at the 64× magnification for stained embryos.

To examine the effect of DAPT and cpd E on Notch signaling, embryos treated with different concentrations of DAPT or cpd E were stained by whole mount *in situ *hybridization using a *her6 *probe (Fig 5). The expression level of Notch downstream target gene *her6 *correlates to the levels of Notch signaling, i.e., a loss of *her6 *staining corresponds to an inhibition of γ-secretase mediated Notch signaling. We have focused on the specific effect of γ-secretase inhibitors on Notch signaling in brain region.

In DMSO-treated embryos, *her6 *expression was mainly clustered in the ventral midbrain and ventral hindbrain (Fig. 5). In the presence of 50 μM DAPT, the *her6 *expression was significantly reduced or disappeared in most areas, reflecting a strong inhibition of γ-secretase activity. When embryos were treated with a lower concentration of DAPT at 5 μM, staining of *her6 *started to appear in those areas found in DMSO-treated embryos. Embryos treated with 0.5 μM DAPT showed a very similar staining pattern to the control embryos. Interestingly, cpd E showed a weaker effect on the expression levels of *her6*. There was a reduction of *her6 *staining in those embryos that were treated with highest testing doses of cpd E. When the *her6 *staining is linked to morphological alterations (Fig. 5), the level of reduction in Notch signaling is closely linked with the severity of phenotypes that was observed in these zebrafish, i.e., the curvature of the tails.

## Discussion

Our knowledge of γ-secretase components distinguishing different substrates provides a molecular basis for the modulation of γ-secretase complex. Nicastrin has been found to interact with both APP and Notch and is involved in substrate recognition and interaction [36]. An artificial elongation of the Pen-2 N-terminus leads to an increased Aβ_42 _production [69], indicating that Pen-2 might function as a modulator to influence the γ-secretase cleavage of APP. Identification of a key regulator of γ-secretase complex TMP21 further suggests that cleavage of APP and Notch could be distinguished and modulated [70]. While the development of γ-secretase inhibitors is one of the major directions for AD therapeutics, completely blocking the γ-secretase-mediated proteolytic process of about 50 substrates interferes with fundamental steps in many biological functions. Therefore, identifying γ-secretase modulators that only block the cleavage of APP, but not other substrates is ideal. Different from earlier studies that have identified NSAIDs and Gleevec for specific blockage of Aβ production without affecting the γ-secretase cleavage of Notch, the current study has provided a systematic approach to identify γ-secretase inhibitors to modulate the γ-secretase cleavage of APP and Notch separately.

We have analyzed two potent γ-secretase inhibitors DAPT and cpd E using different quantification methods to determine the pharmacological profile of blocking the cleavage of APP and Notch. The range of inhibition concentrations vary among these methods. However, the effective inhibitory concentrations for Notch cleavage were always found to be higher than those concentrations for APP cleavage. In a conventional *in vitro *γ-secretase activity assay, 0.1 μM of cpd E completely blocked Aβ generation from the cleavage of substrate APP C100, and only had minor effect on Notch cleavage and NICD generation. Cpd E selectively inhibited the γ-secretase cleavage of APP at low concentrations, i.e., from 0.1 nM to 10 nM. However, at the same concentrations, we found that DAPT did not inhibit the γ-secretase cleavage of APP and Notch (Fig. 1C). When higher concentration of DAPT was used in our *in vitro *γ-secretase activity assay, a partial inhibition of Notch cleavage was observed, in contrast to an almost complete inhibition of APP cleavage. Therefore, DAPT selectively blocked the γ-secretase cleavage of APP at higher concentration compared to compound E. When cpd E or DAPT were applied to HEK293 cells that expressed the substrate NotchΔE, we found that both compounds were more potent in blocking Aβ generation than NICD production. DAPT at concentrations of 1 μM or higher reduced Notch cleavage to about 50% in both *in vitro *γ-secretase activity assay (Fig. 1B) and cell culture based assay (Fig. 2B). Cpd E at 0.1 μM reduced Notch cleavage to ~50% in both systems. For the γ-secretase cleavage of APP, DAPT was able to inhibit the levels of Aβ to 50% at the concentration of 1 μM in vitro and ~0.5 μM in cultured cells, respectively. Compound E, on the other hand, was able to reduce the levels of Aβ to 50% at the concentrations of 1 nM and 5 nM in two systems. Therefore, DAPT and cpd E showed similar potencies in cultured cells and in vitro γ-secretase activity assay. The level of NICD inhibition was consistent with the reduced expression of Luciferase gene driven by a Notch target gene promoter containing three Su(H) binding sequences. Using two previously reported chimeric cDNA constructs expressing APP-m-NOTCH or APP-α-NOTCH, cpd E showed much higher EC_50_'s for lowering the levels of Nβ* derived from the cleavage of APP-m-NOTCH and APP-α-NOTCH. Finally, the expression levels of Notch target gene *her6 *in a whole animal zebrafish, as measured by *in situ *hybridization, were correlated with the dose-dependent phenotypic effect of DAPT. The effect of cpd E was less obvious and hence, consistent with a less reduction of *her6 *expression.

Previous studies have applied similar compounds to differentiate their effect on the γ-secretase cleavage of Notch and APP, and some showed selective inhibition of Aβ production without Notch phenotypes in animals [71]. Lewis et al. have used a set of compounds for the test, and some of these compounds (like compound 1) have similar structures to DAPT [72]. Using cultured cells to test the potencies of different compounds, they found that Notch and APP cleavages cannot be easily dissected apart [72]. We have used additional methods to determine the inhibition profile of DAPT and cpd E, including in vivo animal based assays. In cultured cells expressing NotchΔE or chimeric APP-Notch proteins, cpd E was more effective in inhibiting APP than Notch substrate. DAPT showed similar effect in cultured cells and in an in vitro γ-secretase activity assay. Both γ-secretase inhibitors DAPT and cpd E are believed to interact with the core component of the γ-secretase complex, PS. Mutation of two aspartate residues in PS1 leads to a complete loss of function for γ-secretase which suggests that these two aspartates may constitute the active site of γ-secretase [27]. Both aspartyl protease transition state mimic and non-transition-state γ-secretase inhibitor could specifically bind the N- and C-terminal fragments of PS1 [73,46,57]. The binding of the γ-secretase inhibitor to PS1 NTF/CTF could be then competitively suppressed by the presence of cpd E [57]. DAPT was found to specifically interact with the C-terminal region of PS1 [59]. Studies that use helical peptide inhibitors to block the γ-secretase complex suggest that a docking and an active site exist for the γ-secretase complex, and that the docking site might be located at the PS subunit interface, a site very close to the active site [74]. It is not clear whether different concentrations of DAPT and cpd E may affect the docking site in a way that differentiate the binding of APP and Notch to the γ-secretase complex.

Both DAPT and cpd E have been used to treat animals. DAPT was specifically tested in zebrafish [61]. Zebrafish have a highly conserved γ-secretase complex. Both zebrafish PS1 (Psen1) and the PS2 homolog (Psen2) are expressed during the segmentation and later stages [75-77]. Nicastrin has been identified in the zebrafish genome, and only one copy of Psen1, Psen2, Pen-2, and Aph-1 gene has been found [30]. Once the highly similar zebrafish γ-secretase complex is inhibited by DAPT, somitogenesis is severely affected leading to curved tails, a phenotype well-characterized for altered Notch signaling [61]. In this study, a dose dependent effect of DAPT on zebrafish phenotypes was observed, and a curvature of zebrafish tail was found in embryos treated with 50 μM of DAPT. Although the EC_50 _of DAPT for inhibiting Notch signaling was much lower in cultured cells (1–10 μM, Fig. 2), it is not surprising that a high concentration of DAPT was necessary to induce a phenotype in a whole animal (50 μM, Fig. 4). For cpd E, the highest concentration used to treat embryos was 50 μM compared to an EC_50 _that was below 0.1 μM for the inhibition of NICD generation in cultured cells (Fig. 2). For both DAPT and cpd E, there is no data on pharmacokinetics, pharmacodynamics and ADME of these two compounds in zebrafish. While both cpd E and DAPT are cell permeable, a lack of dramatic phenotypic alteration in embryos treated with 50 μM cpd E could be best explained by a slightly reduced expression level of *her6 *gene. This indicates that Notch signaling was not significantly perturbed at this concentration of cpd E in a whole animal.

Administration of cpd E into guinea pig resulted in a dose-dependent inhibition of brain cortical γ-secretase activity and correspondingly, decreases in plasma, CSF and brain Aβ levels [78]. Treatment of a transgenic mouse expressing human familial AD linked V717F APP with DAPT also leads to a dose-dependent, acute decrease in brain Aβ [79]. Treatment of AD patients with another γ-secretase inhibitor, LY450139 dihydrate, reduces plasma Aβ40. This compound was well tolerated in these patients [80-82]. Therefore, modulated inhibition of γ-secretase is feasible in human subjects, and potent inhibitors used at appropriate doses appear to be promising in preventing the progression of Aβ pathology.

## Conclusion

Our measurement of Aβ and Notch-Aβ-like peptides from chimeric APP proteins could be used for efficient cell-based screening of γ-secretase modulators. These modulators could be tested by *in vitro *γ-secretase activity assay. The *in vivo *test results presented here of these compounds in a vertebrate zebrafish further validate our quantitative methods to differentiate their selectivity for APP, Notch and potentially other γ-secretase substrates.

## Methods

### *In Vitro *γ-Secretase Activity Assay

The *E. coli *generated APP- and Notch-based, 100-residue γ-secretase substrates C100-Flag and N100-Flag were purified as previously described [65]. C100-Flag substrate contains an initiating methionine, the 99 C-terminal residues of APP that start at the α-secretase site, and a Flag tag. N100-Flag substrate contains a similar initiating methionine, 99 amino acids that start at the TACE cleavage site, and a Flag tag. The membrane vesicles were solubilized in 1% CHAPSO-HEPES and diluted in a final concentration of 0.2% CHAPSO-HEPES. Phosphatidylethanolamine (PE) and phosphatidylcholine (PC) were added to the final concentration of 0.02% PE and 0.08% PC. After adding DMSO or test compounds to the solubilized γ-secretase complex, substrate C100-Flag or N100-Flag was added to the mixture, then followed by incubation at 37°C for 4 hours [65,83]. Two compounds have been used in this study, compound E (cpd E), {(S,S)-2-[2-(3,5-Difluorophenyl)-acetylamino]-N-(1-methyl-2-oxo-5-phenyl-2,3-dihydro-1H-benzo [e] [1,4] diazepin-3-yl)-propionamide} [57] and DAPT {N-[N-(3,5-Difluorophenacetyl-L-alanyl)]-S-phenylglycine *t*-Butyl Ester} [79]. Cpd E was provided by Dr. Michael Wolfe.

### ELISAs and Antibodies

Sandwich ELISAs for Aβ were performed as described [84]. The capture antibodies, 2G3 (to Aβ residues 33–40) and 4G8 (to Aβ residues 17–24), were used for Aβ40 and Aβ total species, respectively. The detecting antibodies were biotinylated 82E1 (to Aβ residues 1–16) for Aβ1–40/total or biotinylated 266 for Aβx-40 species. The use of mid-region and C-terminal capturing antibodies and N-terminal detecting antibody for chimeric "Notch-Aβ-like" peptide (Nβ*) has been documented [68]. The combination of several capture/detecting antibodies are use to measure Aβ and Nβ* derived from different precursors. The capture antibody 2G3 and detecting antibody 82E1 were used for measuring Nβ* from APP-α-Notch expressing cells. Since the APP-α-NOTCH is the fusion protein with its juxtamembrane portion of the APP ectodomain (the α-secretase cleavage region) replaced by the corresponding sequence in Notch, the epitopes in APP sequence could still be recognized by 2G3 (C-terminus) and 82E1 (N-terminus). The capture antibody 4G8 and detecting antibody 82E1 were used for measuring Nβ* from APP-m-Notch expressing cells. Since the APP-m-NOTCH is the fusion protein with its transmembrane domain (TMD) replaced by the Notch TMD, the epitopes in APP sequence could be recognized by 4G8 (mid-region before TMD) and 82E1 (N-terminus). Antibody 82E1 was purchased from Immuno-Biological Laboratories, Inc., Minneapolis, MN. Antibody 4G8 was purchased from Signet Laboratories, Inc., Dedham, MA. Antibody 1744 that specifically detect the N-terminus of NICD was purchased from Cell Signaling Technology, Danvers, MA.

### cDNA constructs for cell based γ-secretase activity assay

The cDNA construct NotchΔE has a c-myc tag and is a truncated Notch molecule that is an immediate substrate for γ-secretase cleavage to generate Notch intracellular domain (NICD) [85]. Two chimeric cDNA constructs express APP with (APP-m-NOTCH), or else the juxtamembrane portion of the APP ectodomain (the α-secretase cleavage region) replaced by the corresponding sequence in Notch (APP-α-NOTCH). These cDNA constructs were provided by Dr. Dennis Selkoe [68]. Hes-1 reporter construct (Hes-Luc) was generated by insertion three of Su(H) binding sequence

5'-AGGTTCTCACTGTGGGGTAAGAAGGTTCTCACAGTGGGGTAAGAGGTTCTCACAGTC in the pGL3-pro luciferase reporter vector (Promega, Madison, WI). The final assemble is similar to a previously reported Notch reporter construct [86].

Human embryonic kidney (HEK) 293 cells stably expressing Swedish mutant human APP695 were transfected with different cDNA constructs and maintained in 200 μg/ml G418 (Invitrogen, Carlsbad, CA). Transfected cells were treated with two γ-secretase inhibitors cpd E or DAPT for 8 hr. Conditioned media were collected for ELISA, and cell lysates were analyzed by Western blot as described [87]. Cells co-transfected with Hes-Luc and NotchΔE were treated with compounds followed by the measurement of luciferase activity (Luciferase Assay System, Promega, Madison, WI).

### Zebrafish Embryo Treatment

Zebrafish embryos were raised and staged according to Kimmel, et al. [88]. Compounds were dissolved in egg water at various final concentrations, and 0.5% DMSO was used as a negative control. Prior to the treatment at 24 hour post fertilization, embryos were de-chorionated manually. Embryos were placed in a 24-well plate (5–6 embryos/well) and treated with the compound containing egg water. Embryos were incubated at 28°C, and photographic images were taken at 2 days and 4 days post fertilization (dpf).

### Microscope Imaging

Compound-treated embryos were observed under an OLYMPUS SZX12 microscope. For examination, embryos were removed from the compound containing medium and placed into 0.4% tricane (3-amino benzoic acidethylester, Sigma, St. Louis, MO) solution. Upon anesthetizing, embryos were placed in 3% methylcellulose for positioning and images were recorded with OLYMPUS Q-COLOR3 camera. Images were taken at the 40× magnification for embryos at 2 and 4 dpf.

### *In situ *Hybridization

*In situ *hybridization of compound-treated embryos was carried out at 2 dpf according to standard protocols [89] using the *her6 *probe. Single-stranded RNA probes against *her6 *were synthesized from a cDNA clone (provided by Dr. P Raymond, University of Michigan, Ann Arbor, MI) using T7 RNA polymerase after linearization by restriction digest. The probe was then labeled with digoxigenin-UTP (Roche, Basel, Switzerland). At least 10 to 20 embryos were examined for each experiment. Images were taken at 64× magnification for stained embryos.

## Abbreviations

AD: Alzheimer's disease; Aβ: amyloid β protein; APP: amyloid precursor protein; Abl: Abelson leukemia; cpd E: compound E; dpf: days post fertilization; EC: effective concentration; HEK: human embryonic kidney; hpf: hours post fertilization; Nβ*: Notch-Aβ-like; NICD: Notch intracellular domain; PS: Presenilin; TMD: transmembrane domain; WB: Western blot.

## Competing interests

The authors declare that they have no competing interests.

## Authors' contributions

TY participated in the design of the study and carried out biochemical studies, DA carried out the zebrafish assays, YG and CAS provided reagents and intellectual contribution to the *in vitro *γ-secretase activity assay, WX conceived of the study, participated in its design, and drafted the manuscript. All authors read and approved the final manuscript.

## References

[B1] Selkoe DJ (2004). Alzheimer disease: mechanistic understanding predicts novel therapies. Ann Intern Med.

[B2] Haass C, Schlossmacher M, Hung AY, Vigo-Pelfrey C, Mellon A, Ostaszewski B, Lieberburg I, Koo EH, Schenk D, Teplow D (1992). Amyloid b-peptide is produced by cultured cells during normal metabolism. Nature.

[B3] Shoji M, Golde TE, Ghiso J, Cheung TT, Estus S, Shaffer LM, Cai X, McKay DM, Tintner R, Frangione B (1992). Production of the Alzheimer amyloid b protein by normal proteolytic processing. Science.

[B4] De Strooper B, Annaert W, Cupers P, Saftig P, Craessaerts K, Mumm JS, Schroeter EH, Schrijvers V, Wolfe MS, Ray WJ (1999). A presenilin-1-dependent gamma-secretase-like protease mediates release of Notch intracellular domain. Nature.

[B5] Okochi M, Steiner H, Fukumori A, Tanii H, Tomita T, Tanaka T, Iwatsubo T, Kudo T, Takeda M, Haass C (2002). Presenilins mediate a dual intramembranous gamma-secretase cleavage of Notch-1. Embo J.

[B6] Fortini ME (2002). Gamma-secretase-mediated proteolysis in cell-surface-receptor signalling. Nat Rev Mol Cell Biol.

[B7] Kopan R, Goate A (2002). Aph-2/Nicastrin: an essential component of gamma-secretase and regulator of Notch signaling and Presenilin localization. Neuron.

[B8] Taniguchi Y, Kim SH, Sisodia SS (2003). Presenilin-dependent "gamma-secretase" processing of deleted in colorectal cancer (DCC). J Biol Chem.

[B9] Ni CY, Murphy MP, Golde TE, Carpenter G (2001). gamma-Secretase Cleavage and Nuclear Localization of ErbB-4 Receptor Tyrosine Kinase. Science.

[B10] Lee HJ, Jung KM, Huang YZ, Bennett LB, Lee JS, Mei L, Kim TW (2002). Presenilin-dependent gamma-secretase-like intramembrane cleavage of ErbB4. J Biol Chem.

[B11] Marambaud P, Shioi J, Serban G, Georgakopoulos A, Sarner S, Nagy V, Baki L, Wen P, Efthimiopoulos S, Shao Z (2002). A presenilin-1/gamma-secretase cleavage releases the E-cadherin intracellular domain and regulates disassembly of adherens junctions. Embo J.

[B12] Marambaud P, Wen PH, Dutt A, Shioi J, Takashima A, Siman R, Robakis NK (2003). A CBP binding transcriptional repressor produced by the PS1/epsilon-cleavage of N-cadherin is inhibited by PS1 FAD mutations. Cell.

[B13] Lammich S, Okochi M, Takeda M, Kaether C, Capell A, Zimmer A-K, Edbauer D, Walter J, Steiner H, Haass C (2002). Presenilin dependent intramembrane proteolysis of CD44 leads to the liberation of its intracellular domain and the secretion of an Abeta-like peptide. J Biol Chem.

[B14] Murakami D, Okamoto I, Nagano OKY, Tomita T, Iwatsubo T, De Strooper B, Yumoto E, Saya H (2003). Presenilin-dependent gamma-secretase activity mediates the intramembranous cleavage of CD44. Oncogene.

[B15] May P, Reddy YK, Herz J (2002). Proteolytic processing of low density lipoprotein receptor-related protein mediates regulated release of its intracellular domain. J Biol Chem.

[B16] Kim D, Ingano L, Kovacs D (2002). Nectin-1a, an immunoglobulin-like receptor involved in the formation of synapses, is a substrate for presenilin/g-secretase-like cleavage. J Biol Chem.

[B17] Ikeuchi T, Sisodia S (2003). The Notch ligands, Delta1 and Jagged2, are substrates for presenilin-dependent "gamma-secretase" cleavage. J Biol Chem.

[B18] Meyer E, Strutz N, Gahring LCRS (2003). Glutamate Receptor Subunit 3 Is Modified by Site-specific Limited Proteolysis Including Cleavage by {gamma}-Secretase. J Biol Chem.

[B19] Scheinfeld MH, Ghersi E, Laky K, Fowlkes BJ, D'Adamio L, Roncarati R, Sestan N, Berechid BE, Lopez PA, Meucci O (2002). Processing of beta-amyloid precursor-like protein-1 and -2 by gamma-secretase regulates transcription. J Biol Chem.

[B20] Walsh DM, Fadeeva JV, LaVoie MJ, Paliga K, Eggert S, Kimberly WT, Wasco W, Selkoe DJ (2003). gamma-Secretase cleavage and binding to FE65 regulate the nuclear translocation of the intracellular C-terminal domain (ICD) of the APP family of proteins. Biochemistry.

[B21] Eggert S, Paliga K, Soba P, Evin G, Masters CL, Weidemann A, Beyreuther K (2004). The proteolytic processing of the amyloid precursor protein gene family members APLP-1 and APLP-2 involves alpha -, beta -, gamma -, and epsilon -like cleavages. Modulation of APLP-1 processing by N-glycosylation. J Biol Chem.

[B22] Kanning KC, Hudson M, Amieux PS, Wiley JC, Bothwell M, Schecterson LC (2003). Proteolytic processing of the p75 neurotrophin receptor and two homologs generates C-terminal fragments with signaling capability. J Neurosci.

[B23] Schulz JG, Annaert W, Vandekerckhove J, Zimmermann P, De Strooper B, David G (2003). Syndecan 3 intramembrane proteolysis is presenilin/gamma-secretase-dependent and modulates cytosolic signaling. J Biol Chem.

[B24] Wilhelmsen K, Geer P van der (2004). Phorbol 12-myristate 13-acetate-induced release of the colony-stimulating factor 1 receptor cytoplasmic domain into the cytosol involves two separate cleavage events. Mol Cell Biol.

[B25] Kuhn PH, Marjaux E, Imhof A, De Strooper B, Haass C, Lichtenthaler SF (2007). Regulated intramembrane proteolysis of the interleukin-1 receptor II by alpha-, beta-, and gamma-secretase. J Biol Chem.

[B26] Xia W, Wolfe M (2003). Intramembrane proteolysis by presenilin and presenilin-like proteases. J Cell Sci.

[B27] Wolfe MS, Xia W, Ostaszewski BL, Diehl TS, Kimberly WT, Selkoe DJ (1999). Two transmembrane aspartates in presenilin-1 required for presenilin endoproteolysis and g-secretase activity. Nature.

[B28] Yu G, Nishimura M, Arawaka S, Levitan D, Zhang L, Tandon A, Song YQ, Rogaeva E, Chen F, Kawarai T (2000). Nicastrin modulates presenilin-mediated notch/glp-1 signal transduction and betaAPP processing. Nature.

[B29] Chen F, Yu G, Arawaka S, Nishimura M, Kawarai T, Yu H, Tandon A, Supala A, Song Y, Rogaeva E (2001). Nicastrin binds to membrane-tethered Notch. Nat Cell Biol.

[B30] Francis R, McGrath G, Zhang J, Ruddy D, Sym M, Apfeld J, Nicoll M, Maxwell M, Hai B, Ellis MC (2002). aph-1 and pen-2 are required for Notch pathway signaling, g-secretase cleavage of bAPP and presenilin protein accumulation. Dev Cell.

[B31] Lee S, Shah S, Li H, Yu C, Han W, Yu G (2002). Mammalian APH-1 Interacts with Presenilin and Nicastrin, and is Required for Intramembrane Proteolysis of APP and Notch. J Biol Chem.

[B32] Siman R, Velji J (2003). Localization of presenilin-nicastrin complexes and gamma-secretase activity to the trans-Golgi network. J Neurochem.

[B33] Shirotani K, Edbauer D, Capell ASJ, Steiner H, Haass C (2003). gamma-Secretase Activity Is Associated with a Conformational Change of Nicastrin. J Biol Chem.

[B34] Alves da Costa  C, Mattson MP, Ancolio K, Checler F (2003). The C-terminal fragment of presenilin 2 triggers p53-mediated staurosporine-induced apoptosis, a function independent of the presenilinase-derived N-terminal counterpart. J Biol Chem.

[B35] Li T, Ma G, Cai H, Price DL, Wong PC (2003). Nicastrin Is Required for Assembly of Presenilin/gamma-Secretase Complexes to Mediate Notch Signaling and for Processing and Trafficking of beta-Amyloid Precursor Protein in Mammals. J Neurosci.

[B36] Shah S, Lee SF, Tabuchi K, Hao YH, Yu C, LaPlant Q, Ball H, Dann CE, Sudhof T, Yu G (2005). Nicastrin functions as a gamma-secretase-substrate receptor. Cell.

[B37] Goutte C, Tsunozaki M, Hale VA, Priess JR (2002). APH-1 is a multipass membrane protein essential for the Notch signaling pathway in Caenorhabditis elegans embryos. Proc Natl Acad Sci.

[B38] Crystal A, Morais VA, Pierson TC, Pijak DS, Carlin D, Lee VM, Doms RW (2003). Membrane topology of gamma-secretase component PEN-2. J Biol Chem.

[B39] Baulac S, LaVoie MJ, Kimberly WT, Strahle J, Wolfe MS, Selkoe DJ, Xia W (2003). Functional gamma-secretase complex assembly in Golgi/trans-Golgi network: interactions among presenilin, nicastrin, Aph1, Pen-2, and gamma-secretase substrates. Neurobiol Dis.

[B40] De Strooper B (2003). Aph-1, Pen-2, and Nicastrin with Presenilin generate an active gamma-Secretase complex. Neuron.

[B41] Hu Y, Fortini M (2003). Different cofactor activities in gamma-secretase assembly: evidence for a nicastrin-Aph-1 subcomplex. J Cell Biol.

[B42] Kimberly W, LaVoie M, Ostaszewski BL, Ye W, Wolfe MS, Selkoe DJ (2003). Gamma-secretase is a membrane protein complex comprised of presenilin, nicastrin, Aph-1, and Pen-2. Proc Natl Acad Sci USA.

[B43] Luo WJ, Wang H, Li H, Kim BS, Shah S, Lee HJ, Thinakaran G, Kim TW, Yu G, Xu H (2003). PEN-2 and APH-1 coordinately regulate proteolytic processing of presenilin 1. J Biol Chem.

[B44] Takasugi N, Tomita T, Hayashi I, Tsuruoka M, Niimura M, Takahashi Y, Thinakaran G, Iwatsubo T (2003). The role of presenilin cofactors in the gamma-secretase complex. Nature.

[B45] Edbauer D, Winkler E, Regula JT, Pesold B, Steiner H, Haass C (2003). Reconstitution of gamma-secretase activity. Nat Cell Biol.

[B46] Esler WP, Kimberly WT, Ostaszewski BL, Diehl TS, Moore CL, Tsai JY, Rahmati T, Xia W, Selkoe DJ, Wolfe MS (2000). Transition-state analogue inhibitors of gamma-secretase bind directly to presenilin-1. Nat Cell Biol.

[B47] Li Y-M, Xu M, Lai M-T, Huang Q, Castro JL, DiMuzio-Mower J, Harrison T, Lellis C, Nadin A, Neduvelli JG (2000). Photoactivated g-secretase inhibitors directed to the active site covalently label presenilin 1. Nature.

[B48] Weggen S, Eriksen JL, Sagi SA, Pietrzik CU, Golde TE, Koo EH (2003). Abeta42-lowering nonsteroidal anti-inflammatory drugs preserve intramembrane cleavage of the amyloid precursor protein (APP) and ErbB-4 receptor and signaling through the APP intracellular domain. J Biol Chem.

[B49] Weggen S, Eriksen JL, Das P, Sagi SA, Wang R, Pietrzik CU, Findlay KA, Smith TE, Murphy MP, Bulter T (2001). A subset of NSAIDs lower amyloidogenic Abeta42 independently of cyclooxygenase activity. Nature.

[B50] Gasparini L, Rusconi L, Xu H, del Soldato P, Ongini E (2004). Modulation of beta-amyloid metabolism by non-steroidal anti-inflammatory drugs in neuronal cell cultures. J Neurochem.

[B51] Qin W, Ho L, Pompl PN, Peng Y, Zhao Z, Xiang Z, Robakis NK, Shioi J, Suh J, Pasinetti GM (2003). Cyclooxygenase (COX)-2 and COX-1 potentiate beta-amyloid peptide generation through mechanisms that involve gamma-secretase activity. J Biol Chem.

[B52] Yan Q, Zhang J, Liu H, Babu-Khan S, Vassar R, Biere AL, Citron M, Landreth G (2003). Anti-inflammatory drug therapy alters beta-amyloid processing and deposition in an animal model of Alzheimer's disease. J Neurosci.

[B53] Eriksen JL, Sagi SA, Smith TE, Weggen S, Das P, McLendon DC, Ozols VV, Jessing KW, Zavitz KH, Koo EH (2003). NSAIDs and enantiomers of flurbiprofen target gamma-secretase and lower Abeta 42 in vivo. J Clin Invest.

[B54] Weggen S, Eriksen JL, Sagi SA, Pietrzik CU, Ozols V, Fauq A, Golde TE, Koo EH (2003). Evidence that nonsteroidal anti-inflammatory drugs decrease amyloid beta 42 production by direct modulation of gamma-secretase activity. J Biol Chem.

[B55] Netzer WJ, Dou F, Cai D, Veach D, Jean S, Li Y, Bornmann WG, Clarkson B, Xu H, Greengard P (2003). Gleevec inhibits beta-amyloid production but not Notch cleavage. Proc Natl Acad Sci USA.

[B56] Milano J, McKay J, Dagenais C, Foster-Brown L, Pognan F, Gadient R, Jacobs RT, Zacco A, Greenberg B, Ciaccio PJ (2004). Modulation of notch processing by gamma-secretase inhibitors causes intestinal goblet cell metaplasia and induction of genes known to specify gut secretory lineage differentiation. Toxicol Sci.

[B57] Seiffert D, Bradley JD, Rominger CM, Rominger DH, Yang F, Meredith JE, Wang Q, Roach AH, Thompson LA, Spitz SM (2000). Presenilin-1 and -2 are molecular targets for gamma-secretase inhibitors. J Biol Chem.

[B58] Fraering PC, LaVoie MJ, Ye W, Ostaszewski BL, Kimberly WT, Selkoe DJ, Wolfe MS (2004). Detergent-dependent dissociation of active gamma-secretase reveals an interaction between Pen-2 and PS1-NTF and offers a model for subunit organization within the complex. Biochemistry.

[B59] Morohashi Y, Kan T, Tominari Y, Fuwa H, Okamura Y, Watanabe N, Sato C, Natsugari H, Fukuyama T, Iwatsubo T (2006). C-terminal fragment of presenilin is the molecular target of a dipeptidic gamma-secretase-specific inhibitor DAPT (N-[N-(3,5-difluorophenacetyl)-L-alanyl]-S-phenylglycine t-butyl ester). J Biol Chem.

[B60] Fraering PC, Ye W, LaVoie MJ, Ostaszewski BL, Selkoe DJ, Wolfe MS (2005). gamma-Secretase substrate selectivity can be modulated directly via interaction with a nucleotide-binding site. J Biol Chem.

[B61] Geling A, Steiner H, Willem M, Bally-Cuif L, Haass C (2002). A gamma-secretase inhibitor blocks Notch signaling in vivo and causes a severe neurogenic phenotype in zebrafish. EMBO Rep.

[B62] Jiang YJ, Brand M, Heisenberg CP, Beuchle D, Furutani-Seiki M, Kelsh RN, Warga RM, Granato M, Haffter P, Hammerschmidt M (1996). Mutations affecting neurogenesis and brain morphology in the zebrafish, Danio rerio. Development.

[B63] van Eeden FJ, Granato M, Schach U, Brand M, Furutani-Seiki M, Haffter P, Hammerschmidt M, Heisenberg CP, Jiang YJ, Kane DA (1996). Mutations affecting somite formation and patterning in the zebrafish, Danio rerio. Development.

[B64] Petit A, Pasini A, Alves Da Costa C, Ayral E, Hernandez JF, Dumanchin-Njock C, Phiel CJ, Marambaud P, Wilk S, Farzan M (2003). JLK isocoumarin inhibitors: selective gamma-secretase inhibitors that do not interfere with notch pathway in vitro or in vivo. J Neurosci Res.

[B65] Esler WP, Kimberly W, Ostaszewski B, Ye W, Diehl T, Selkoe D, Wolfe MS (2002). Activity dependent isolation of the presenilin-g-secretase complex reveals nicastrin and a g substrate. Proc Natl Acad Sci.

[B66] Campbell W, Wolfe M, Xia W, Xia W, Xu H (2005). Assays for Amyloid Precursor Protein g-Secretase Activity. Amyloid Precursor Protein, A Practical Approach.

[B67] Ray WJ, Yao M, Nowotny P, Mumm J, Zhang W, Wu JY, Kopan R, Goate AM (1999). Evidence for a physical interaction between presenilin and Notch. Proc Natl Acad Sci USA.

[B68] Zhang J, Ye W, Wang R, Wolfe MS, Greenberg BD, Selkoe DJ (2002). Proteolysis of chimeric beta-amyloid precursor proteins containing the Notch transmembrane domain yields amyloid beta-like peptides. J Biol Chem.

[B69] Isoo N, Sato C, Miyashita H, Shinohara M, Takasugi N, Morohashi Y, Tsuji S, Tomita T, Iwatsubo T (2007). Abeta42 overproduction associated with structural changes in the catalytic pore of gamma-secretase: common effects of Pen-2 N-terminal elongation and fenofibrate. J Biol Chem.

[B70] Chen F, Hasegawa H, Schmitt-Ulms G, Kawarai T, Bohm C, Katayama T, Gu Y, Sanjo N, Glista M, Rogaeva E (2006). TMP21 is a presenilin complex component that modulates gamma-secretase but not epsilon-secretase activity. Nature.

[B71] Best JD, Smith DW, Reilly MA, O'Donnell R, Lewis HD, Ellis S, Wilkie N, Rosahl TW, Laroque PA, Boussiquet-Leroux C (2007). The novel gamma secretase inhibitor N-[cis-4-[(4-chlorophenyl)sulfonyl]-4-(2,5-difluorophenyl)cyclohexyl]-1,1,1-trifl uoromethanesulfonamide (MRK-560) reduces amyloid plaque deposition without evidence of notch-related pathology in the Tg2576 mouse. J Pharmacol Exp Ther.

[B72] Lewis HD, Perez Revuelta BI, Nadin A, Neduvelil JG, Harrison T, Pollack SJ, Shearman MS (2003). Catalytic site-directed gamma-secretase complex inhibitors do not discriminate pharmacologically between Notch S3 and beta-APP cleavages. Biochemistry.

[B73] Li YM, Xu M, Lai MT, Huang Q, Castro JL, DiMuzio-Mower J, Harrison T, Lellis C, Nadin A, Neduvelil JG (2000). Photoactivated gamma-secretase inhibitors directed to the active site covalently label presenilin 1. Nature.

[B74] Kornilova AY, Bihel F, Das C, Wolfe MS (2005). The initial substrate-binding site of gamma-secretase is located on presenilin near the active site. Proc Natl Acad Sci USA.

[B75] Leimer U, Lun K, Romig H, Walter J, Grunberg J, Brand M, Haass C (1999). Zebrafish (Danio rerio) Presenilin Promotes Aberrant Amyloid b-Peptide Production and Requires a Critical Aspartate Residue for Its Function in Amyloidogenesis. Biochem.

[B76] Groth C, Nornes S, McCarty R, Tamme R, Lardelli M (2002). Identification of a second presenilin gene in zebrafish with similarity to the human Alzheimer's disease gene presenilin2. Dev Genes Evol.

[B77] Nornes S, Groth C, Camp E, Ey P, Lardelli M (2003). Developmental control of Presenilin1 expression, endoproteolysis, and interaction in zebrafish embryos. Exp Cell Res.

[B78] Grimwood S, Hogg J, Jay MT, Lad AM, Lee V, Murray F, Peachey J, Townend T, Vithlani M, Beher D (2005). Determination of guinea-pig cortical gamma-secretase activity ex vivo following the systemic administration of a gamma-secretase inhibitor. Neuropharmacology.

[B79] Dovey HF, John V, Anderson JP, Chen LZ, de Saint Andrieu P, Fang LY, Freedman SB, Folmer B, Goldbach E, Holsztynska EJ (2001). Functional gamma-secretase inhibitors reduce beta-amyloid peptide levels in brain. J Neurochem.

[B80] Siemers ER, Dean RA, Friedrich S, Ferguson-Sells L, Gonzales C, Farlow MR, May PC (2007). Safety, tolerability, and effects on plasma and cerebrospinal fluid amyloid-beta after inhibition of gamma-secretase. Clin Neuropharmacol.

[B81] Siemers ER, Quinn JF, Kaye J, Farlow MR, Porsteinsson A, Tariot P, Zoulnouni P, Galvin JE, Holtzman DM, Knopman DS (2006). Effects of a gamma-secretase inhibitor in a randomized study of patients with Alzheimer disease. Neurology.

[B82] Fleisher AS, Raman R, Siemers ER, Becerra L, Clark CM, Dean RA, Farlow MR, Galvin JE, Peskind ER, Quinn JF (2008). Phase 2 safety trial targeting amyloid beta production with a gamma-secretase inhibitor in Alzheimer disease. Arch Neurol.

[B83] Xia W, Xu H (2004). Amyloid Precursor Protein, A Practical Approach.

[B84] Johnson-Wood K, Lee M, Motter R, Hu K, Gordon G, Barbour R, Khan K, Gordon M, Tan H, Games D (1997). Amyloid precursor protein processing and A beta42 deposition in a transgenic mouse model of Alzheimer disease. Proc Natl Acad Sci USA.

[B85] Ray WJ, Yao M, Mumm J, Schroeter E, Saftig P, Wolfe M, Selkoe D, Kopan R, Goate AM (1999). Cell surface presenilin-1 participates in the g-secretase-like cleavage of Notch. J Biol Chem.

[B86] Berechid BE, Kitzmann M, Foltz DR, Roach AH, Seiffert D, Thompson LA, Olson RE, Bernstein A, Donoviel DB, Nye JS (2002). Identification and characterization of presenilin-independent Notch signaling. J Biol Chem.

[B87] Xia W, Zhang J, Perez R, Koo EH, Selkoe DJ (1997). Interaction between amyloid precursor protein and presenilins in mammalian cells: implications for the pathogenesis of Alzheimer disease. Proc Natl Acad Sci USA.

[B88] Kimmel CB, Ballard WW, Kimmel SR, Ullmann B, Schilling TF (1995). Stages of embryonic development of the zebrafish. Dev Dyn.

[B89] Thisse C, Thisse B, Schilling TF, Postlethwait JH (1993). Structure of the zebrafish snail1 gene and its expression in wild-type, spadetail, and no tail mutant embryos. Development.

